# IMMUNEPOTENT CRP induces DAMPS release and ROS-dependent autophagosome formation in HeLa and MCF-7 cells

**DOI:** 10.1186/s12885-020-07124-5

**Published:** 2020-07-13

**Authors:** Ana Carolina Martínez-Torres, Alejandra Reyes-Ruiz, Kenny Misael Calvillo-Rodriguez, Karla Maria Alvarez-Valadez, Ashanti C. Uscanga-Palomeque, Reyes S. Tamez-Guerra, Cristina Rodríguez-Padilla

**Affiliations:** 1 Universidad Autonoma de Nuevo Leon, Facultad de Ciencias Biologicas, Laboratorio de Inmunologia y Virologia, San Nicolás de los Garza, Mexico; 2 Longeveden, SA de CV, Monterrey, Mexico

**Keywords:** Autophagy, DAMPs, Bovine dialyzable leukocyte extract, ROS, Immunotherapy, Transfer factor

## Abstract

**Background:**

IMMUNEPOTENT CRP (ICRP) can be cytotoxic to cancer cell lines. However, its widespread use in cancer patients has been limited by the absence of conclusive data on the molecular mechanism of its action. Here, we evaluated the mechanism of cell death induced by ICRP in HeLa and MCF-7 cells.

**Methods:**

Cell death, cell cycle, mitochondrial membrane potential and ROS production were evaluated in HeLa and MCF-7 cell lines after ICRP treatment. Caspase-dependence and ROS-dependence were evaluated using QVD.oph and NAC pre-treatment in cell death analysis. DAMPs release, ER stress (eIF2-α phosphorylation) and autophagosome formation were analyzed as well. Additionally, the role of autophagosomes in cell death induced by ICRP was evaluated using SP-1 pre-treatment in cell death in HeLa and MCF-7 cells.

**Results:**

ICRP induces cell death, reaching CC_50_ at 1.25 U/mL and 1.5 U/mL in HeLa and MCF-7 cells, respectively. Loss of mitochondrial membrane potential, ROS production and cell cycle arrest were observed after ICRP CC_50_ treatment in both cell lines, inducing the same mechanism, a type of cell death independent of caspases, relying on ROS production. Additionally, ICRP-induced cell death involves features of immunogenic cell death such as P-eIF2α and CRT exposure, as well as, ATP and HMGB1 release. Furthermore, ICRP induces ROS-dependent autophagosome formation that acts as a pro-survival mechanism.

**Conclusions:**

ICRP induces a non-apoptotic cell death that requires an oxidative stress to take place, involving mitochondrial damage, ROS-dependent autophagosome formation, ER stress and DAMPs’ release. These data indicate that ICRP could work together with classic apoptotic inductors to attack cancer cells from different mechanisms, and that ICRP-induced cell death might activate an immune response against cancer cells.

## Background

Among the different types of cancer, breast and cervical cancer remain the principal causes of women death worldwide [1]. Main treatments consist of surgical removal of the tumor, chemotherapy, radiation therapy, hormonal therapy, and immunotherapy. However, these treatments still have limited success, and the development of new therapies to improve existing ones is a major challenge.

IMMUNEPOTENT CRP (ICRP), a bovine dialyzable leukocyte extract (DLE) obtained from disintegrated spleen, is cytotoxic to several cancer cell lines, including those from lung cancer [2] cervical cancer [3] and breast cancer [4, 5], while sparing noncancerous cells [6]. In murine melanoma, it prevented cell growth and diminished VEGF release [7]. In the cervical cancer cell lines HeLa and SiHa, and the non-small cell lung cancer cell lines A549, and A427, it induced cell cycle arrest and caspase-independent but ROS-dependent cell death [2, 3]. Additionally, its administration promoted a decrease in tumor volume and an increase in the survival of mice bearing 4 T1 tumors without visibly affecting vital organs, or hematological and biochemical parameters [8]. Additionally, ICRP induced immunogenic cell death (ICD) alone or in combination with oxaliplatin in the murine model B16F10 [9]; this immunogenicity of cancer cell death relies on the antigenicity of the neoantigens expressed by dead cancer cells and the release of damage-associated molecular patterns (DAMPs) such as calreticulin (CRT), ATP and HMGB1 [10]. Until today, every ICD inductor causes endoplasmic reticulum (ER) stress, which implies several cellular processes as eIF2α phosphorylation (P-eIF2α) and exposure of chaperone proteins like CRT [11]. Besides ER stress, production of reactive oxygen species (ROS) is an essential component that instigates the intracellular danger-signalling pathways that govern ICD. ROS and other reactive species are the main intracellular signal transducers sustaining autophagy, thus, several studies have shown an autophagy-ROS dependence for the release of DAMPs [12, 13].

Autophagy is a primary survival mechanism activated in cells subjected to stress. However, if cellular stress continues, autophagy often becomes associated with features of cell death. This dual role of autophagy has been associated with the resistance of cancer cells to treatments (as a pro-survival process) or the induction of cell death (as a pro-death process) depending on the stimulus. Moreover, autophagy can be dispensable for the induction of cell death but required for its immunogenicity [14, 15].

The purpose of this study was to analyze the molecular pathways by which ICRP exerts its cytotoxicity. We used HeLa and MCF-7 cell lines to further characterize its mechanism of cytotoxicity evaluating cell cycle, mitochondrial membrane potential, caspase and ROS dependence for cell death, autophagosome formation, eIF2-α phosphorylation, DAMPs release and the role of autophagy in the mechanism of ICRP-induced cell death.

## Methods

### Cell culture

Human cervix adenocarcinoma HeLa (ATCC® CCL-2™) and human breast adenocarcinoma MCF-7 (ATCC® HTB-22™) cells were obtained from the American Type Culture Collection (2015), mycoplasma tested (last test August 2019), and maintained in a humidified incubator containing 5% CO_2_ at 37 °C. Cells were cultured in DMEM-F12 supplemented with 10% fetal bovine serum (FBS) and 1% penicillin-streptomycin (Life Technologies, Grand Island, NY), and were routinely grown in plastic tissue-culture dishes (Life Sciences, Corning, NY).

Peripheral blood mononuclear cells (PBMC) extraction.

After obtaining written informed consent, PBMC were isolated from healthy donors by density gradient centrifugation with Ficoll-Paque™ PLUS (GE Healthcare, Chicago, Ilinois, USA) and maintained at 4X10^6^ cells/mL in cell culture plates at 37 °C in 5% CO_2_ atmosphere, using RPMI 1640 medium (GIBCO Thermofisher, Waltham, Massachusetts, USA) supplemented with 1 μg/mL amphotericin B, 1 μg/mL penicillin and 2.5X10^− 3^ μg/mL streptomycin (GIBCO Thermofisher, Waltham, Massachusetts, USA) and 10% of FBS (GIBCO Thermofisher, Waltham, Massachusetts, USA). The study was approved by the Institutional Ethics Committee at the Universidad Autonoma de Nuevo León, College of Biological Sciences.

### Cell death analysis

Cell death was determined by staining cells with 2.5 μg/mL APC Annexin V (BD Pharmingen, San Jose, CA) and 0.5 μg/mL propidium iodide (PI) (Sigma-Aldrich, ST. Louis, MO). In brief, 5 × 10^4^ cells were seeded in 24-well plates and were incubated with IMMUNEPOTENT CRP for 24 h, with or without pre-incubation with QVD.oph (10 μM), N-acetyl-L-cysteine (NAC) (5 mM) or Spautin-1 (Sp-1) (15 μM). Cells were then recuperated, washed with PBS (Phosphate-buffered saline) and then resuspended in 100 μl of binding buffer (10 mM HEPES/ NaOH pH 7.4, 140 mM NaCl, 2.5 mM CaCl_2_). Finally, cells were stained, incubated at 4 °C for 20 min and assessed with BD Accury C6 flow cytometer (Becton Dickinson, Franklin Lakes, NJ). The results were analyzed using FlowJo Software (LLC, Ashland, OR).

### Mitochondrial membrane potential analysis

Mitochondrial membrane potential was measured using 500 nM TMRE (Sigma-Aldrich, ST. Louis, MO). In brief, 5 × 10^4^ cells in 24-well dishes were incubated with or without IMMUNEPOTENT CRP (CC_50_) for 24 h. Cells were then recuperated, washed with PBS, stained, incubated at 37 °C for 30 min, and measured by flow cytometry as described above.

### ROS production analysis

ROS generation was measured using 2.5 μM DCFDA (Thermo Fisher Scientific, Waltham, MA). In brief, 5 × 10^4^ cells in 24-well dishes were incubated with IMMUNEPOTENT CRP (CC_50_) for 24 h, with or without pre-incubation with NAC. Cells were then recuperated, washed with PBS, stained, incubated at 37 °C for 30 min, and measured by flow cytometry as mention.

### Cell cycle analysis

Cell cycle distributions were determined by PI (Sigma-Aldrich, ST. Louis, MO) staining. 2 × 10^5^ cells in 6-well dishes were incubated with ICRP for 24 h. Cells were then washed with PBS and fixed in 70% ethanol. Cells were washed with PBS, then incubated with 50 μg/mL PI and simultaneous 50 μg/mL RNase (Sigma-Aldrich, ST. Louis, MO) treatment at 37 °C for 30 min. Cell DNA contents were measured by flow cytometry as explained above.

### eIF2α phosphorylation analysis

For this assay, 2 × 10^5^ cells were plated in 12-well dishes and were incubated with ICRP for 18 h. Cell were collected and fixed (eBioscience™ Foxp3 / Transcription Factor Fixation/Permeabilization) for 1 h at 4 °C, washed with 2%-FACS Buffer (PBS 1x and 2% FBS), centrifuged twice at 2000 rpm during 20 min, suspended in 10%-FACS Buffer (PBS 1x and 10% FBS) and incubated for 30 min in shaken. Then, anti-EIF2S1 (phospho S51) antibody [E90] (Abcam, ab32157) (1:100) was added and incubated for 2 h and washed twice. Next, goat anti-rabbit IgG H&L (Alexa Fluor® 488) (Abcam, ab150077) (1:200) was added and incubated for 1 h in darkness. Finally, cells were washed and eIF2α phosphorylation was measured by flow cytometry as described above.

For confocal microscopy 1.5 × 10^5^ cells were planted on cover slides into 12-well dishes, treated with ICRP (CC_50_) and incubated for 24 h. Then, cells were washed and fixed with 4% PFA, washed and permeabilized with 0.1% Triton Buffer, washed twice with 2% FACS buffer, and 10%-FACS buffer was added and incubated during 45 min. Next, recombinant anti-EIF2S1 (phospho S51) antibody [E90] (Abcam, ab32157) (1:250) was added, incubated for 2 h and washed thrice. Finally, goat anti-rabbit IgG H&L (Alexa Fluor® 488) (Abcam, ab150077) (1:100) was added, incubated for 30 min in darkness, washed twice and assessed by confocal microscopy (Olympus X70).

### Calreticulin exposure

For this evaluation, 1 × 10^6^ cells were plated, left untreated or treated with ICRP, and incubated for 24 h. Cells were harvested, washed, and stained with Calreticulin-Phycoerythrin (Calreticulin-PE, FMC-75; Enzo Life Science, Farmingdale, NY) antibody (1:1000) in 2%-FACS buffer. After 1 h in darkness at room temperature (RT), cells were washed and suspended in 100 microliters uL of 2%-FACS buffer to be assessed by flow cytometry as mention before.

For confocal microscopy, 2.5 × 10^5^ cells were plated, and then left untreated (control) or treated with ICRP (CC_50_) and incubated for 24 h. Then, cells were washed with PBS, stained with Calreticulin-PE antibody (2 μg/mL) (Enzo Life Science, Farmingdale, NY) and incubated for 1 h in FACS buffer. Finally, cells were washed twice with PBS and assessed by confocal microscopy (Olympus X70).

### ATP release assay

For this, 1 × 10^6^ cells/mL were treated with ICRP for 24 h. Supernatants were used to assess extracellular ATP by a luciferase assay (ENLITEN kit, Promega, Madison, WI) following the manufacturer’s instructions. Bioluminescence was assessed in the Synergy HT microplate reader using the Software Gen5 (BioTek, Winooski, VT) at 560 nm.

### High-mobility group box 1 release assay

Supernatants of untreated and ICRP-treated cells (1 × 10^6^ cells/mL) were used to measure extracellular HMGB1 using an HMGB1 ELISA kit (BioAssay ELISA kit human; US Biological Life Science, Salem, MA), following the manufacturer’s instructions. Absorbance was assessed in the Synergy HT microplate reader using the Software Gen5 (BioTek, Winooski, VT) at 450 nm.

### Cell morphology assessment

HeLa and MCF-7 cells were cultured in 24-well plates and left untreated or incubated for 24 h with ICRP, with or without pre-incubation with NAC (20 min). After the incubation time, cells were observed in an inverted microscope (NIKON TS100) and pictures were obtained with an Infinity1 (Lumera) camera (10X).

### Autophagosome formation analysis

For this assay, 5 × 10^4^ cells were cultured in 24-well plates (Life Sciences) and left untreated or incubated for 24 h with ICRP, with or without pre-incubation with Sp-1 or NAC. Then, cells were recuperated, washed with PBS, stained with CYTO-ID Autophagy Detection Kit (Enzo Life Science, Farmingdale, NY) and measured by flow cytometry as explained above.

### Statistical analysis

The results presented here represent the mean of at least three independent experiments done in triplicate (mean ± SD). Statistical analysis was done using paired student T-test, and the statistical significance was defined as *p* < 0.05. The data was analyzed using GraphPad Prism (GraphPad Software, San Diego, CA, USA).

## Results

### IMMUNEPOTENT CRP induces cell death in HeLa and MCF-7 cells through cell cycle arrest, loss of mitochondrial membrane potential and ROS generation

**Fig. 1:**
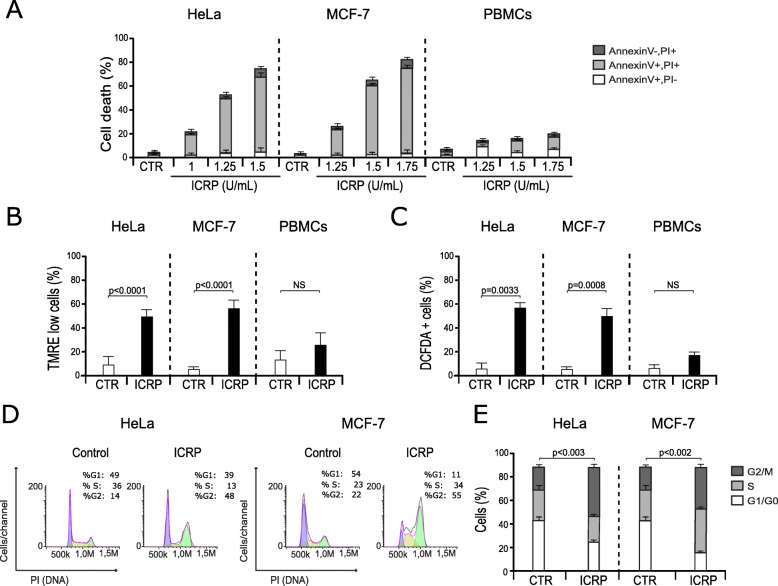
IMMUNEPOTENT CRP induces RCD through mitochondrial and cell cycle alterations in HeLa and MCF-7 cells. **a**. Cell death was measured by flow cytometry through Annexin-V and PI staining and graphed, in HeLa (left) and MCF-7 cells (center), and PBMC (right) treated with different concentrations of ICRP for 24 h. **b**. Loss of ΔΨm induced by ICRP was measured by flow cytometry using TMRE staining in HeLa (1.25 U/mL, 24 h) (left), MCF-7 cells (1.5 U/mL, 24 h) (center), and PBMC (1.5 U/mL, 24 h) (right). **c**. ROS levels were measured by flow cytometry through DCFDA staining in HeLa (1.25 U/mL, 24 h) (left), MCF-7 cells (1.5 U/mL, 24 h) (center), and PBMC (1.5 U/mL, 24 h) (right). **d**. Schematic representation of changes in cellular DNA content measured by flow cytometry through PI staining in HeLa (1.25 U/mL, 24 h) (left) and MCF-7 cells (1.5 U/mL, 24 h) (right). **e**. The results obtained as in (D) were analyzed using Flowjo software and graphed. The graphs represent the means (± SD) of triplicates of at least three independent experiments

ICRP induces regulated cell death in HeLa, SiHa, A549 and A427 cells [2, 3], while sparing noncancerous cells [6], however its effect on breast cancer derived-MCF-7 cell line, has not been assessed. Thus, to determine the effect of ICRP in MCF-7 cells, we evaluated cell death induced by different doses of ICRP after 24 h of treatment using HeLa cells and healthy-donor derived PBMC as a control. ICRP induced cell death in a concentration-dependent manner, in both cell lines, after 24 h of treatment (Fig. 1a), while PBMC were only slightly affected. Cell death was characterized by double-positive Annexin-V and PI staining, as previously reported for cervical cancer [3] and lung cancer cells [2]. In HeLa cells, ICRP provoked cell death (Annexin-V and/or PI staining) in 30% of the cells at 1 U/mL dose, reaching 50% at 1.25 U/mL and increasing near to 90% at 1.5 U/mL. In MCF-7 cells, ICRP induced a slight cell death at 1.25 U/mL (less than 20% of the cells), and at 1.5 U/mL it induced cell death in 50% of the cells, reaching 80% at 1.75 U/mL. On the other hand, ICRP induced a slight cell death induction at 1.25 U/mL and at 1.5 U/mL (less than 20% of the cells), reaching 20% of cell death at 1.75 U/mL in PBMC (Fig. 1a).

Moreover, it is known that mitochondria play a central role in cell death signaling, as mitochondrial dysfunction leads to ROS generation which has been associated with many types of cell death [16]. Thus, loss of mitochondrial membrane potential and ROS production were evaluated in HeLa, MCF-7, and PBMC after ICRP CC_50_ treatment for 24 h. As expected, loss of mitochondrial membrane potential (Fig. 1b) and ROS generation (Fig. 1c) were observed in 50% of the HeLa and MCF-7 cells after treatment in both cell lines, whereas in PBMC we could observe a slight and non-significant loss of mitochondrial membrane potential (Fig. 1b) and ROS production (Fig. 1c). Because we observed that ICRP did not generate a significant affectation in PBMC, further studies were continued using only HeLa and MCF-7 cell lines.

ICRP is known to induce cell cycle arrest in HeLa cells in a time-dependent manner, reaching the maximum accumulation of cells in G2/M phase after 24 h of treatment [3]. Here we evaluated the cell cycle of MCF-7 cells after the treatment with ICRP using HeLa cells as a control. As seen in Fig. 1d and e, ICRP effectively induces cell cycle arrest in the G2/M phase in both cell lines after 24 h of treatment. Additionally, ICRP also induces cell cycle arrest in S phase in MCF-7 cells (Fig. 1d, e).

### IMMUNEPOTENT CRP induces caspase-independent cell death but in a ROS-dependent manner in HeLa and MCF-7 cells

**Fig. 2:**
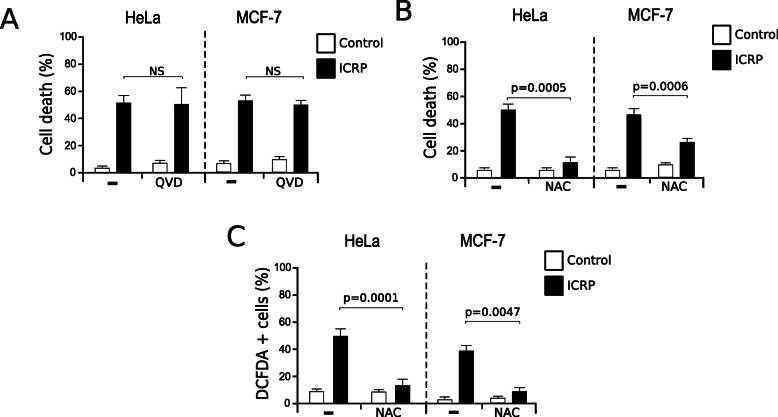
IMMUNEPOTENT CRP induces caspase-independent cell death in a ROS-dependent manner in HeLa and MCF-7 cells. **a**. Cell death was evaluated in HeLa (left) and MCF-7 cells (right) left alone or pretreated with QVD.oph before ICRP treatment (24 h). **b**. Effect on cell death of HeLa (left) and MCF-7 cells (right) left alone or pretreated with NAC before ICRP treatment (24 h). **c**. ROS production was measured in HeLa (left) and MCF-7 cells (right) left alone or pretreated with NAC before ICRP treatment (24 h), through DCFDA staining. The charts represent the means (± SD) of triplicates of at least three independent experiments

Once we confirmed that the principal cell death features induced by ICRP in HeLa cells were maintained in breast cancer cells, we next wondered if the cell death was also caspase-independent, as previously shown in HeLa cells [3]. To determine this, a pan-caspase inhibitor, QVD.oph, was used before treatment with ICRP. As shown in Fig. 2a, ICRP-mediated cell death was independent of caspase activation in HeLa and MCF-7 cells.

Then, as ROS generation has been associated with caspase-independent types of cell death [17], the antioxidant NAC was used to determine if ROS were playing a role in ICRP-induced cell death. NAC was able to inhibit ICRP-mediated cell death (Fig. 2b) by reducing ROS production in both cell lines (Fig. 2c).

### IMMUNEPOTENT CRP induces eIF2α phosphorylation and DAMPS release in HeLa and MCF-7 cells

**Fig. 3:**
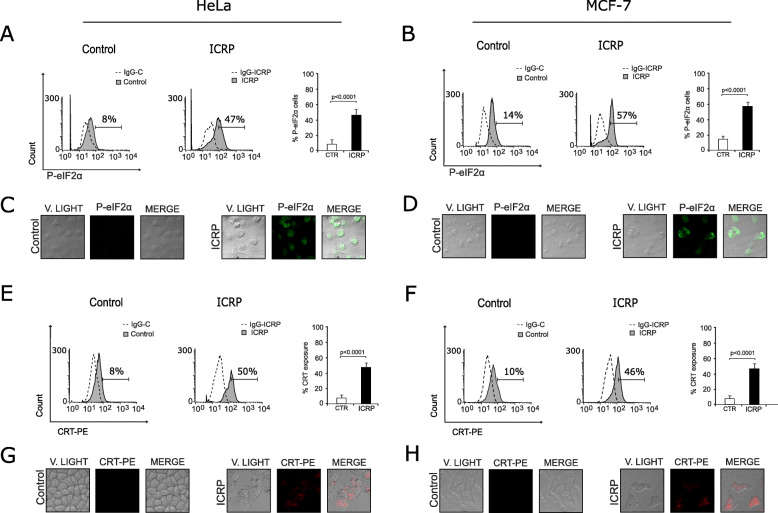
IMMUNEPOTENT CRP induces eIF2α phosphorylation and CRT exposure in HeLa and MCF-7 cells. **a**, **b** eIF2α phosphorylation (P-eIF2α) in HeLa (**a**) and MCF-7 (**b**) cells measured by flow cytometry. Negative controls, with IgG isotype antibodies, are shown in dotted, and in gray Control (without treatment) and ICRP treatment. **c**, **d**. Confocal microscopy representation of P-eIF2α in HeLa (**c**) and MCF-7 (**d**) cells. Representative diagrams of surface CRT detection in HeLa (**e**) and MCF-7 (**f**) cells using FACS. Negative controls, with IgG isotype antibodies, are shown in dotted, and in gray Control (without treatment) and ICRP treatment. CRT exposure in HeLa (**g**) and MCF-7 (**h**) cells was observed after treatment with ICRP by CRT-PE staining and visualized by confocal microscopy. Graphs represent the means (± SD) of triplicates of at least three independent experiments

Recently, our research group found that ICRP induced the release of several DAMPs (CRT, ATP, HSP70, HSP90 and HMGB1), and ICD in B16F10 murine melanoma cells [9]. However, these features have not been assessed in human cancer cells. Considering that one of the first steps in the induction of ICD is the activation of an ER stress response, which involves the phosphorylation of eIF2α (P-eIF2α) [11]; this parameter was evaluated in human cancer cell lines by flow cytometry after 18 h of treatment. As shown in Fig. 3a and b, ICRP was able to induce eIF2α phosphorylation in 47 and 57% of HeLa and MCF-7 cells treated with ICRP, respectively; this was confirmed by confocal microscopy (Fig. 3c and Fig. 3d). Because DAMPs exposure and release has been implicated in cell death involving ER stress [10], the next step was to evaluate if treatment with ICRP could induce the exposure or release of the principal DAMPs in the human cancer cell lines HeLa and MCF-7.

**Fig. 4:**
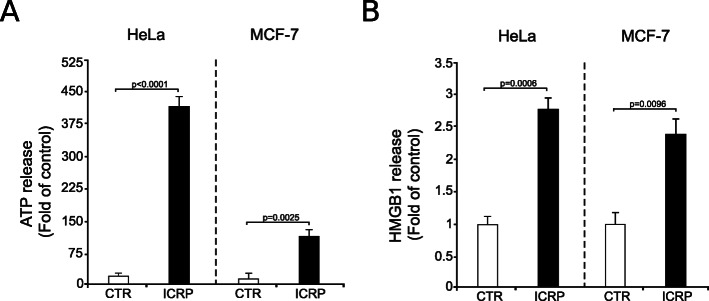
IMMUNEPOTENT CRP induces ATP and HMGB1 release in HeLa and MCF-7 cells. Cells were treated with ICRP (HeLa: 1.25 U/mL; MCF-7: 1.5 U/mL) for 24 h, then 100 μL supernatant of each sample was taken to measure **a**. ATP release through bioluminescence detection or **b**. HMGB1 release by ELISA. Graphs shown are means (± SD) of triplicates of three independent experiments

First, CRT exposure was assessed by flow cytometry and the results showed that 50% of HeLa (Fig. 3e) and MCF-7 (Fig. 3f) cells treated with ICRP exposed CRT, which was confirmed by confocal microscopy (Fig. 3g and Fig. 3h). Furthermore, as observed in Fig. 4, ICRP induced 393- and 114-fold ATP release, and 2.7- and 2.4-fold HMBG1 release in HeLa and MCF-7 cells, respectively, as compared with untreated control. These results indicate that ICRP induces ER stress and DAMPs release in cervical and breast cancer cell lines.

### IMMUNEPOTENT CRP induces ROS-dependent autophagosome formation in HeLa and MCF-7 cells

**Fig. 5:**
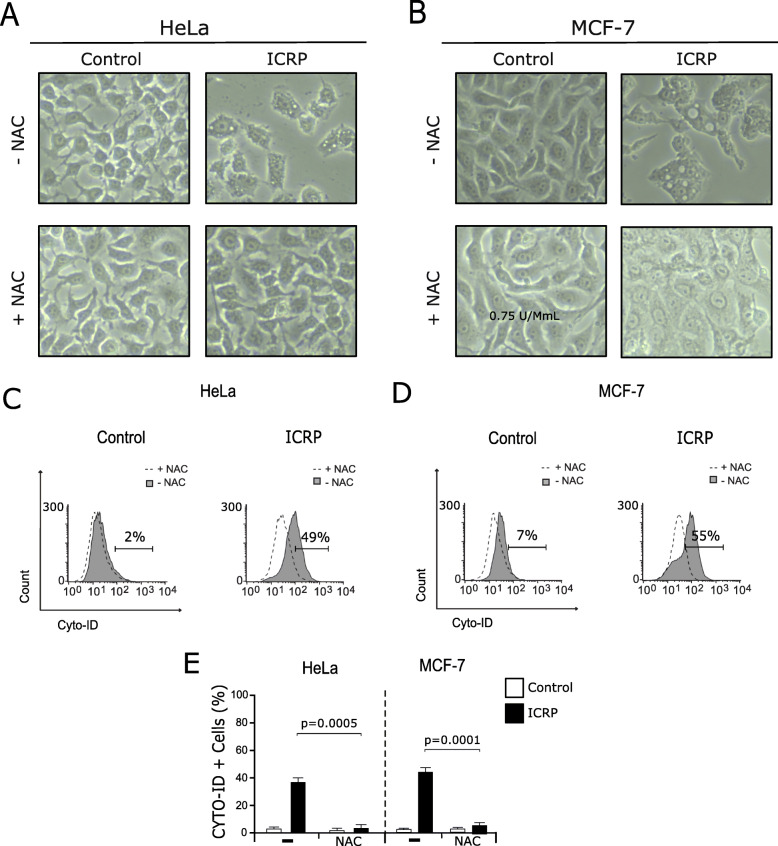
IMMUNEPOTENT CRP induces autophagosome formation in HeLa and MCF-7 cells. **a**. HeLa, and **b**. MCF-7 cells were left alone or pretreated with NAC before ICRP treatment (24 h), and observed by optical microscopy (10X). **c**, **d**. Autophagosome formation was determined by flow cytometry through CYTO-ID staining in HeLa (**c**) and MCF-7 cells (**d**) that were left alone or pretreated with NAC before ICRP treatment (24 h). **e**. The results were analyzed using Flowjo software and graphed. Graphs shown are means (± SD) of triplicates of three independent experiments

ROS production and autophagosome formation have been related with the release of DAMPs. For this reason, evaluation of both characteristics was assessed in cervical and breast cancer cell lines. Morphological assessment of cells treated with ICRP indicated the presences of vacuoles in HeLa (Fig. 5a) and MCF-7 (Fig. 5b) cells. Additionally, intracellular formation of vacuoles depended of ROS production in cells treated with ICRP (Fig. 5a and Fig. 5b), thus, the next step was to evaluate if autophagy was taking place after ICRP treatment and if this was dependent of ROS production. We demonstrated that ICRP effectively induced autophagosome formation in approximately 40% of Hela (Fig. 5c) and 50% of MCF-7 (Fig. 5d) cells. Furthermore, autophagosome formation was ROS-dependent, as the use of the antioxidant NAC completely inhibits autophagosomes in cells treated with ICRP (Fig. 5c-e).

### IMMUNEPOTENT CRP induces pro-survival autophagosomes in HeLa and MCF-7 cells

**Fig. 6:**
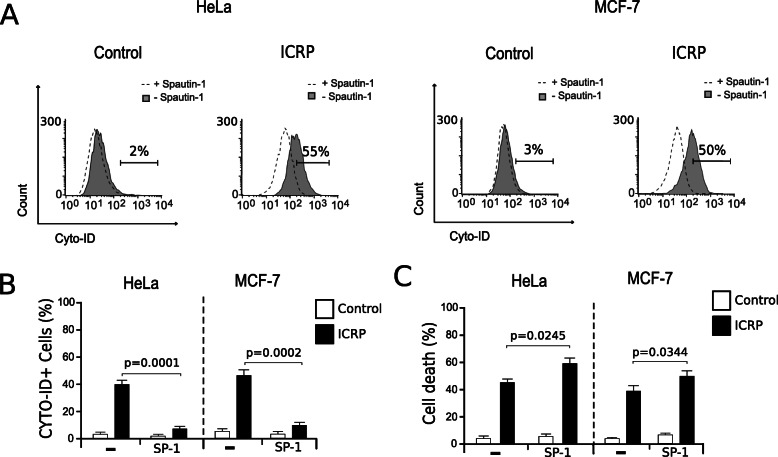
IMMUNEPOTENT CRP induces prosurvival autophagosome formation in HeLa and MCF-7 cells. **a**. Autophagosome formation was determined by flow cytometry through CYTO-ID staining in HeLa (left) and MCF-7 cells (right) that were left alone or pretreated with SP-1 before ICRP treatment (24 h) **b**. The results were analyzed using FlowJo software and graphed **c**. Cell death was determined by flow cytometry in HeLa (left) and MCF-7 cells (right) that were left alone or pretreated with SP-1 before ICRP treatment (24 h). Graphs shown are means (± SD) of triplicates of three independent experiments

Autophagy can be pro-survival or pro-death [18]. Indeed, there is a type of regulated cell death that relies on the autophagic machinery (or components) called “autophagic cell death” which is characterized by being caspase-independent [14, 17]. We observed autophagosome formation and caspase-independent cell death after ICRP treatment in HeLa and MCF-7 cells, the next question was whether the mechanism of death induced by ICRP relied on autophagy. Therefore, the autophagic inhibitor Spautin-1 (SP-1) was used to analyze if autophagosome formation was pro-survival or pro-death. For this purpose, autophagosome formation was assessed with or without SP-1 pre-treatment. In Fig. 6, SP-1 was able to completely inhibit autophagosome formation in HeLa (Fig. 6a) and MCF-7 cells (Fig. 6b). Then, the role of autophagosomes in the cell death induced by ICRP was evaluated. Results show that cell death was not inhibited with SP-1 (Fig. 6c), furthermore, cell death significatively augmented when autophagy was inhibited. Through these results we can conclude that ICRP induces pro-survival autophagosome formation in both cell lines.

## Discussion

Cancer is a heterogeneous disease, and one of the challenges of cancer treatment is that each type of cancer has different molecular features. Here, we use two different cell lines, human cervix adenocarcinoma HeLa and human breast adenocarcinoma MCF-7 cells, and show that ICRP induces cell death, loss of mitochondrial membrane potential, ROS production and cell cycle arrest. Both cell lines exhibit the same mechanism, that is to say, a type of caspase-independent cell death that relies on ROS production. The observation of a conserved cell death mechanism in both cell lines has been reported in studies with other agents. For instance, Khazaei et al. demonstrated that broadleaf wild leek (*Allium atroviolaceum*) bulb extract induces cell death in HeLa and MCF-7 cells, sharing some features of the cell death mechanism induced by this treatment, such as Bcl-2 downregulation, DNA degradation and caspase activation on both cell lines [19]. On the other hand, Martinez-Torres et al., found that chitosan gold nanoparticles (CH-AuNPs) induce cell death in HeLa and MCF-7 cells though different cell death mechanisms. In HeLa cells they observed that the cell death induced by CH-AuNPs was dependent of caspase activation, whereas it was caspase independent in MCF-7 cells. However, they also proved that ROS production seems to be a conserved feature of the cell death mechanism induced by CH-AuNPs [20]. In another study, Green tea polyphenols (GTP) were evaluated in vitro in different cell lines, where they found that MCF-7 cells where more sensitive to the treatment with GTP than HeLa cells [21]. These variable mechanisms observed in HeLa and MCF-7 cells rely on the similarities and differences between the molecular machinery existing on each cell line. Here, we have proved that ICRP could induce a conserved mechanism of cell death in these two cell lines, as observed before in non-small cell lung cancer cell lines (A549 and A427 cells) where ICRP induced DNA degradation, mitochondrial damage, ROS production, and cell death independent of caspases but reliant on ROS production [2]. This indicates that the treatment with ICRP can overcome the mechanisms of cell death resistance existing in different types of cancer cells. However, it is important to mention, that differences in the cell death mechanism induced by ICRP in HeLa and MCF-7 cells could be observed in further analyses.

There is still much to understand about the mechanisms by which ICRP exerts these effects, nevertheless, in this work, we have shown that the cytotoxic effect induced by ICRP in HeLa and MCF-7 cells relies on the increase of ROS production. Interestingly, in our previous work, we observed that, in HeLa cells, the increase of ROS formation is one of the first steps of cell death induced by ICRP, even before caspase activation [3]. This ROS-dependent cell death has been induced by other treatments. For example, Wu et al. demonstrated that the protein kinase C inhibitor, Chelerythrine, could induce cell death through ROS-dependent ER stress in human prostate cancer cells [22]. Furthermore, Kim et al. observed that Resveratrol-induced cell death in ovarian cancer cells was attenuated by the antioxidant NAC, and there was a ROS-dependent decrease of Notch1 signaling on these cells after treatment [23]. However, despite the implication of ROS in cell death, there are some agents that induce cell death without relying in ROS production [24–26].

Additionally, ROS production has been related with the induction of ICD, considering that DAMPs release is either accompanied or triggered by ROS [27]. Recently, our research group found that ICRP induces ICD in a murine melanoma model [9]. Here, we observed that ICRP induces the exposure and release of the principal DAMPs (CRT, ATP and HMBGB1) and eIF2α phosphorylation, a process known as an ER stress indicator and biomarker of ICD [11], in the human cancer cells, HeLa and MCF-7 cells, indicating that ICRP could induce ICD in these models. Caspase-independent ICD has been observed in studies with other treatments, for instance, the CD47-agonist peptide PKHB1-induced caspase-independent and Ca^2+^-dependent cell death, pursing an immunogenic mechanism of cell death in leukemic cells [28, 29]; in addition, Giampazolias et al. demonstrated that in contrast to apoptosis, cells undergoing caspase-independent cell death generated a pro-inflammatory and immunogenic anti-tumour response through the activation of nuclear factor-κB (NF-κB) [30].

Furthermore, recent studies argue that ROS and other reactive species are the main intracellular signal transducers sustaining autophagy, because the ROS scavenger NAC attenuated induced autophagy by citreoviridin [31], hydroxysafflor yellow A-sonodynamic therapy [32], ergosterol peroxide from marine fungus Phoma sp. [33], patulin [34], dimethylaminomicheliolide [35] and many other agents. In this work, we proved that ICRP induces ROS-dependent autophagosome formation in both cell lines. This ROS-Autophagy interplay has been related with the induction of ICD as well, because these processes can play a central role in the exposure and release of DAMPs [12]. ROS production, autophagosome formation, DAMPs release and ER stress have been observed in the induction of ICD by other treatments, including mitoxantrone, doxorubicin, oxaliplatin and photodynamic therapy [10, 27, 36–38]. However, it will be necessary to perform gold-standard vaccination experiments to determine whether ICRP is a bona fide ICD inducer in these cancer models [39].

Moreover, autophagy has been referred to as a double-edge sword because it helps maintain cell homeostasis but, in certain contexts, excessive or sustained cell autophagy may be pro-death [18]. Furthermore, autophagy may not be necessary for the induction of cell death but may be required for its immunogenicity [40], thus, we evaluated the role of autophagosome formation in the mechanism of cell death induced by ICRP, finding that it induces prosurvival autophagosomes in HeLa and MCF-7 cells. This role of autophagy as a cancer cell’s pro-survival response to therapeutics has been observed in many treatments, including trastuzumab [41], epiribicin [42], tamoxifen [43], paclitaxel [44] and radiation [45]. Thus, it will be important to evaluate the role of autophagy in the induction of DAMPs release by ICRP to have a better understanding of the mechanism of action of this treatment.

Here we demonstrate that ICRP is able to induce a selective non-apoptotic cell death that promotes ER-stress and DAMP’s release. These characteristics shed light into the therapeutic potential of the combination of ICRP with traditional chemotherapies, which seems encouraging, as observed in murine melanoma where ICRP increased the immunogenicity of oxaliplatin treatment [9]. Additionally, in the 4 T1 murine model it was observed that ICRP improved the antitumor effects of doxorubicin/cyclophosphamide treatment [8]. Thus, further assessments to describe the cell death mechanism and the potential immunogenic mechanism of the combination of ICRP with classical chemotherapies will undoubtedly straighten its applicability and advantage against cervical and breast cancer.

## Conclusions

Overall our results show that IMMUNEPOTENT CRP induces cell cycle arrest, mitochondrial damage, ROS-dependent autophagosome formation with a pro-survival role, ER stress and DAMPs release, pursing a non-apoptotic cell death, relying on ROS production in HeLa and MCF-7 cells. These data postulate ICRP as a treatment that could execute a conserved mechanism of cell death, in spite of the heterogeneity of cancer cells, opening the gate to the study of the immunogenic potential of ICRP-induced cell death in cervical and breast cancer models.

## Data Availability

The datasets used and/or analysed during the current study are available from the corresponding author on reasonable request.

## Abbreviations

ICRP: IMMUNEPOTENT-CRP
ROS: Reactive oxygen species
RCD: Regulated Cell Death
Ann/PI: Annexin-V-Allocp/ Propidium iodide
ICD: Immunogenic cell death
ER: Endoplasmic Reticulum
DAMPs: Damage-associated molecular patterns
